# Effect of Disulfide Bond Density on the Properties of Polyurethane/Epoxy Interpenetrating Networks

**DOI:** 10.3390/ma18071636

**Published:** 2025-04-03

**Authors:** Gudong You, Xi Li, Kaiwen Ren, Tao Ai, Yanhui Niu

**Affiliations:** School of Materials Science and Engineering, Chang’an University, Xi’an 710061, China; ygd603182@163.com (G.Y.);

**Keywords:** disulfide bond density, damping material, IPNs, self-healing, shape memory

## Abstract

Interpenetrating polymer networks (IPNs) are widely used as damping materials across various industries. However, they are susceptible to issues such as microcracking or fracture over long-term service periods. To address these challenges and improve the long-term performance of IPNs, this research focused on designing and synthesizing self-healing polyurethane (PU)/epoxy (EP) interpenetrating networks (PU/EP-IPNs) enhanced with dynamic disulfide bonds. The incorporation of these bonds significantly enhanced the damping and self-healing properties of the materials. The shape memory performance was evaluated, demonstrating high shape fixation rates of up to 95.0% and exceptional shape recovery rates of up to 99.7%. These results indicate the materials’ ability to revert to their original shape upon heating above the glass transition temperature (*T*_g_). In addition, the effective damping temperature range of the material reached 61.4 °C, and the loss factor was 0.859. This indicates that the enhancement of damping performance is closely related to the increase in disulfide bond density. The formation of the IPN between PU and EP also contributed to improved mechanical and thermomechanical properties. These PU/EP-IPNs exhibit significant potential as innovative damping materials with self-healing capabilities.

## 1. Introduction

Automated machinery and equipment operation aims to enhance production speed and efficiency; however, it also generates substantial noise and vibration, adversely impacting both mechanical equipment and human health. Given the critical nature of this issue, it is essential to adopt appropriate strategies for vibration and noise control [1,2]. In the range of solutions under consideration, damping substances have been shown to possess particular value on account of their capacity to affect the transformation of kinetic energy into heat energy [3], thereby offering an effective solution to the detrimental effects under discussion. These materials have a wide range of applications in a variety of fields [4].

Damping materials are capable of absorbing and attenuating vibrations, impacts, and sound waves. The distinctive molecular configuration of these materials facilitates the efficient absorption of mechanical energy, which is subsequently converted into heat energy for effective dispersion. This functionality not only safeguards structural integrity and mechanical components but also contributes to noise attenuation. In general, four types of damping materials are identified: viscoelastic damping materials, high damping alloy materials, intelligent damping materials, and composite damping materials [5]. Among them, viscoelastic damping materials are particularly favored by researchers due to their ease of processing and shaping, structural designability, and cost-effectiveness.

Viscoelastic damping materials are distinguished by their polymeric nature, exhibiting non-uniform molecular configurations. When not under tension, the molecular chains appear in the form of entangled, long-chain molecules. Since the distance between their ends is much smaller than the entire length of the molecules, these molecules exhibit a unique combination of viscosity and elasticity. Nevertheless, prolonged service can lead to the development of microcracks within the polymer, potentially resulting in material failure and a reduction in lifespan.

The damping factor (ƍ) and the effective damping temperature range (temperature interval where tanƍ ≥ 0.3) are commonly used to evaluate the damping properties of polymers. Ideal damping materials exhibit a high damping factor, indicating excellent energy dissipation capability, with a damping temperature range typically 60–80 °C [6,7,8]. The damping effect is more pronounced when the ambient temperature of the polymer material is aligned with its effective damping temperature range. One-component polymers typically have a relatively low *T*_g_ and an effective damping temperature range of 10–30 °C, limiting their potential applications. Extensive research has been carried out on various strategies to improve damping properties, including the synthesis of copolymers [9,10], polymer blending techniques [11,12,13], and chemical modification approaches [14,15].

The concept of interpenetrating polymer networks (IPNs) was first proposed by Millar in the 1960s, which are polymer alloys formed by a network of two or more crosslinked polymers bonded by a permanent physical entanglement or occasional covalent chemical bond [16]. The “forced intercapacitation” and “synergistic effect” characteristics of the IPN enhance the compatibility between the components, allowing for materials to exhibit both the intrinsic characteristics of the constituent polymers and the improved properties of the polymer blends. The macroscopic homogeneous phase and microscopic multiphase micro-regions within the IPN further improve the peak damping factors and broaden the damping temperature domains.

However, the frequent absorption and energy dissipation in IPNs can result in fatigue, microcracks, and even fractures [17]. If IPNs can be manufactured to possess self-healing capabilities, their service life can be significantly extended [18]. Self-healing polymers containing dynamic covalent bonds are activated by external stimuli to enable the self-repair function [19]. These dynamic covalent bonds are often introduced through chemical reactions, including diselenides [20], disulfide bonds [21], Diels–Alder reactions [22], boronic esters [23], olefin complexation reactions [24], ester-exchange reactions [25], and carbamate/urea transcarbamylation reactions [26,27,28,29]. Recent studies have documented numerous examples of disulfide bonds being introduced into self-healing polymers. Chang et al. [30] developed a transparent polyurethane material with self-healing properties, incorporating disulfide bond chemistry. This was achieved through a two-step synthetic approach. The resulting polymeric network demonstrates dual functionality: crack closure through shape memory effects and intrinsic self-repair capabilities mediated by dynamic disulfide bond exchange reactions. The network demonstrated healing efficiency and elongation at break exceeding 90% and 800%, respectively, and exhibited good healing repeatability and reliability. Wang et al. [31] achieved remarkable mechanical properties and room-temperature self-healing capabilities in silicone rubber through the introduction of a trifunctional crosslinker, “hexamethylene diisocyanate trimer (Tri-HDI)”, into the poly(dimethylsiloxane) linear structure and the subsequent distribution of dynamic disulfide bonds surrounding it. Wu et al. [32] successfully synthesized high-performance epoxy insulating materials with varying disulfide bond contents. A small amount of disulfide bonds was found to enhance properties such as the elongation at break of the epoxy insulating materials. Mechanical damage self-healing tests demonstrated that the epoxy resin containing 0.125 mol of disulfide bonds exhibited the strongest self-repair performance, with a self-healing efficiency of 81.23%. Fu et al. [33] introduced reversible hydrogen bonds and disulfide bonds into polyurethane films while dispersing carbon-coated nickel nanoparticles as fillers into the matrix during the initial polymerization stage. After curing, self-healing polyurethane nanocomposite films (PUHN) were obtained. The results indicated that the introduction of reversible disulfide bonds and the presence of a large number of hydrogen bonds endowed PUHN with excellent self-healing properties, achieving a self-healing efficiency of up to 91.2%. Liu et al. [34] described a polyurethane containing disulfide bonds capped with isocyanate, prepared by curing a poly (vinyl alcohol)-graft-(ε-caprolactone) polymer. This polymer exhibited multiple crosslinked structures and hydrogen bonds, which endowed it with favorable tensile properties and self-healing behavior at moderate temperatures (up to 94% self-healing). However, none of the preceding studies focused on the influence of disulfide bond content or density on the performances of the interpenetrating network.

In a previous study [35], a novel polyurethane/epoxy interpenetrating polymer network (PU/EP-IPN) system was successfully developed through the innovative utilization of 2,2′-diaminodiphenyl disulfide (2-ADPS) as a dual-functional agent, serving simultaneously as a curing agent for the epoxy resin and a chain extender for the polyurethane. The structural characterization and performance evaluation demonstrated that the synthesized materials incorporated dynamic disulfide bonds within their network architecture, consequently exhibiting remarkable damping characteristics. To investigate the effect of disulfide bond density on the properties of interpenetrating networks, this work employs di-octachlorodiphenylamine methane (MOCA) as a partial replacement for 2-ADPS in the synthesis of PU/EP-IPNs with varying disulfide bond densities. The structure and properties of the prepared PU/EP-IPNs were thoroughly characterized using Fourier transform infrared spectroscopy (FT-IR), thermogravimetric analysis (TGA), dynamic mechanical analysis (DMA), scanning electron microscopy (SEM), and tensile tests. The findings demonstrate that an increase in dynamic disulfide bond density markedly enhances the damping performance, expands the effective damping temperature range, and improves the overall mechanical properties of the materials.

## 2. Materials and Methods

### 2.1. Materials

Polyether polyol (POP, analytical grade, Mw = 2000 g·mol^−1^) was obtained from Dow Chemical Company, Shanghai, China. 2,4-tolylene diisocyanate (TDI, 98%) was supplied by Shandong Yantai Wanhua Chemical Group Co. (Yantai, China). Di-octachlorodiphenylamine methane (MOCA, ≥98%) was purchased from Aladdin (Shanghai, China). Bisphenol A epoxy resin (E51, EEW = 184–195 g·mol^−1^) was from Baling Petrochemical Company, Yueyang, China. 2-Aminophenyl disulfide (2-ADPS, 98%) and cashew phenol (99.5%) were purchased from Macklin (Shanghai, China).

### 2.2. Synthesis of PU/EP-IPNs

#### 2.2.1. Synthesis of Polyurethane Prepolymers and Epoxy Resin Precursors

TDI (3.14 g, 18 mmol) and dry POP (12 g, 6 mmol) were thoroughly stirred in order to create a homogeneous mixture. Subsequently, the mixture was subjected to a vacuum oven at 120 °C for 2 h, yielding the polyurethane prepolymer.

The epoxy resin E51 (9 g, 48 mmol) was transferred into a clean beaker, where a quantitative amount of 2-ADPS or MOCA (refer to Table 1 for relevant data) was added. The mixture was then heated and stirred at 120 °C for 30 min. Subsequently, cashew phenol, representing 10% of the total mass, was introduced to the mixture to enhance the degree of interpenetration and miscibility of the system. The epoxy precursor was ultimately obtained through thorough mixing.

#### 2.2.2. Preparation of PU/EP-IPNs

**Figure 1:**
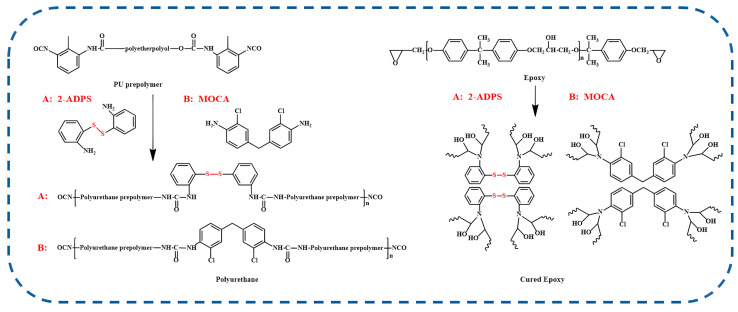
The synthetic route of PU/EP-IPNs.

The polyurethane prepolymer (36 mmol, 10.1 g) was mixed with a specified quantity of the epoxy precursor and stirred for 30 s. The mixture was then poured into a preheated polytetrafluoroethylene mold and cured in a vacuum oven according to a temperature program comprising heating at 80 °C for 16 h and 120 °C for 4 h. The synthetic route of the interpenetrating network is shown in Figure 1.

The cured samples were labeled IPN-PU/EP-SS_x_, where x denoted the percentage of dynamic disulfide bonds of the PU/EP-IPNs and was controlled to be 0%, 50%, and 100%. The samples were labeled as IPN-PU/EP-SS_0_, IPN-PU/EP-SS_0.5_, and IPN-PU/EP-SS_1_, respectively. All samples were kept at room temperature for 148 h, after which they were subjected to structural characterization and performance evaluation. The specific composition of each sample is summarized in Table 1.

### 2.3. Characterization

The chemical structure characterization was performed using an ALPHA-2 Fourier transform infrared spectrometer (Bruker, Berlin, Germany), with spectral acquisition parameters set to a wavenumber range of 4000–400 cm^−1^, spectral resolution at 4 cm^−1^, and 32 accumulated scans. Thermal decomposition behavior was evaluated through thermogravimetric analysis using a Q500 instrument (TA Instruments, New Castle, DE, USA) under nitrogen purge (50 mL min^−1^), with the temperature ramping from 30 to 700 °C at a constant heating rate of 10 °C min^−1^. A DMA Q800 (TA Instruments, New Castle, DE, USA) was employed to evaluate the material’s damping performance, storage modulus, and loss modulus over time at the temperature range from −50 to 150 °C in a fixed frequency of 1 Hz. The samples (30 mm × 5 mm × 1 mm) were subjected to tension mode testing with a temperature increase rate of 3 °C min^−1^. Additionally, stress relaxation experiments were conducted on the samples, which were initially aligned by preloading with a force of 0.1 N and equilibrated for 5 min after reaching each test temperature. Then, the samples were applied to a constant strain of 2% for monitoring.

The tensile properties of the PU/EP-IPNs (160 mm × 20 mm × 1 mm) were assessed using a testing machine (SANS-CMT5105, SSANS Testing Technology Co., Ltd., Shenzhen, China) according to the GB/T1040.3-2006 standard [36]. The experiments were carried out with a speed of 20 mm min^−1^. Morphological characterization of the fracture surfaces of the PU/EP-IPNs was conducted using a Sigma 300 field emission scanning electron microscope (Zeiss, Oberkochen, Germany) operated at 5 kV accelerating voltage, with the specimens subjected to gold sputter coating prior to imaging. The self-healing characteristics were quantitatively assessed by optical microscopy (E31SPM, Touptekphotonics Co., Ltd., Hangzhou, China). The shape memory performance of the IPN-PU/EP-SS_x_ was evaluated in various manners. Specifically, after heating to 80 °C, the IPN-PU/EP-SS_x_ was folded, twisted, stretched, and frozen at room temperature to maintain the temporary shapes. The sample was placed into a hot water bath (80 °C) to record the recovery process. To further analyze the shape memory properties of the PU/EP-IPNs, a U-shaped steel tool with a radius of 15 mm was used. The shape recovery rate (*R*_r_) and shape fixing rate (*R*_f_) were obtained based on the following Equations (1) and (2):

(1) $$R_{f}=\frac{\theta_{\mathrm{fix}}}{\theta_{\max}}$$

(2) $$R_{r}=\frac{\theta_{\mathrm{fix}}-\theta_{\mathrm{rec}}}{\theta_{\mathrm{fix}}}\times 100\%$$

where *θ*_fix_ represents the angle at which the specimen is fixed after bending; *θ*_max_ is the maximum angle at which the specimen is unfolded; and *θ*_rec_ is the angle at which the specimen is returned.

## 3. Results and Discussions

### 3.1. Structural Characterization

**Figure 2:**
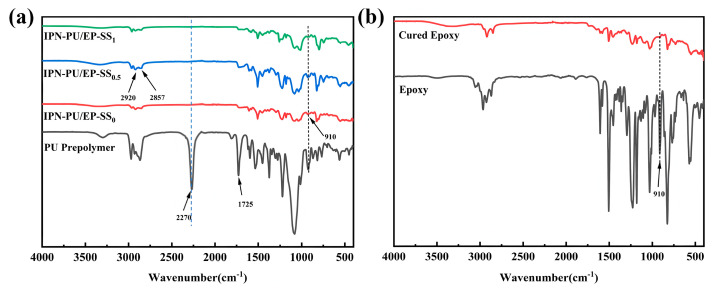
(**a**) FT-IR plots of polyurethane prepolymers and different IPN-PU/EP-SS_x_; (**b**) FT-IR plots of epoxy resin before and after curing.

Fourier transform infrared (FT-IR) spectroscopy was employed to identify the functional groups and characterize the structural features of the synthesized polymers. The FT-IR spectra of the polyurethane prepolymer and IPN-PU/EP-SS_x_ are shown in Figure 2a. The characteristic absorption peaks for the stretching vibrations of -CH_3_ and -CH_2_ are observed at 2857 cm^−1^ and 2920 cm^−1^, respectively. A prominent peak in the polyurethane prepolymer spectrum at 2270 cm^−1^ confirms the presence of free isocyanate groups (-NCO). The FT-IR spectra of the epoxy resin before and after curing are shown in Figure 2b. The characteristic epoxy group absorption at 910 cm^−1^ vanishes in the cured resin spectrum, demonstrating complete ring-opening reactions during curing. The disulfide bonds were not detected in FTIR due to their weak signal intensity [37,38], but their incorporation into the system was indirectly confirmed through the reaction mechanism analysis [39]. During the synthesis of IPN-PU/EP-SS_x_, the –NCO groups of polyurethane reacted with the amino moieties from either MOCA or 2-ADPS, while the epoxy groups underwent ring opening, followed by subsequent bonding with amino groups. Consequently, the successful incorporation of dynamic disulfide bonds reduced the content of both –NCO and epoxy groups in the system, leading to decreased peak intensities at 2270 cm^−¹^ and 910 cm^−¹^. This phenomenon was clearly observed in the FT-IR spectra. The above results demonstrate the successful incorporation of disulfide bonds into the IPN system. The reactions promoted mutual interpenetration of polyurethane and epoxy components, culminating in the establishment of a three-dimensional crosslinked network structure.

### 3.2. Thermal Performance

**Figure 3:**
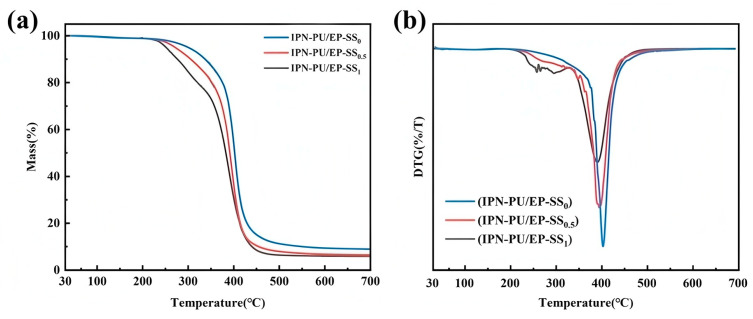
Thermal stability properties of IPN-PU/EP-SS_X_: (**a**) TGA; (**b**) DTG.

A thermogravimetric analysis (TGA) was conducted to evaluate the thermal stability properties of IPN-PU/EP-SS_x_. The TGA and derived thermogravimetric analysis (DTG) curves for IPN-PU/EP-SS_x_ are presented in Figure 3. IPN-PU/EP samples with three different disulfide bond densities demonstrated analogous weight loss patterns across the temperature range of 30 to 700 °C. In the temperature range between 30 and 230 °C, all samples demonstrated a slight thermal weight loss with minimal variation. All samples exhibited an initial decomposition within the temperature range of 230–350 °C. Notably, different initial decomposition temperatures were observed. It can be clearly observed that IPN-PU/EP-SS_0_ exhibited a smaller weight loss in this stage. The polyurethane component of IPN-PU/EP-SS_0_ contains urethane bonds, which possess relatively lower thermal stability. As a result, thermal decomposition occurs within this range, leading to the initial weight loss. In contrast to IPN-PU/EP-SS_0_, the structures of IPN-PU/EP-SS_0.5_ and IPN-PU/EP-SS_1_ incorporate disulfide bonds. Compared to urethane, the lower bond energy and weaker thermal stability of these disulfide bonds are the factors that cause the thermal decomposition of IPN-PU/EP-SS_0.5_ and IPN-PU/EP-SS_1_ to commence earlier, thus resulting in greater weight loss. It has been demonstrated that an increase in the density of disulfide bonds results in a substantial decline in the thermal stability of the material. In the range of 350–400 °C, the weight loss of all samples exhibited a significant increase, predominantly due to the thermal decomposition of the polyurethane hard segment components (isocyanate, polyether polyol) and the epoxy resin. This observation aligns with previously reported trends in weight loss in similar studies [40]. The incorporation of TDI in the polyurethane component and the benzene ring structure of 2-ADPS or MOCA resulted in enhanced thermal stability, as evidenced by the initial thermal decomposition temperatures exceeding 230 °C for all three materials.

### 3.3. Mechanical Properties

**Figure 4:**
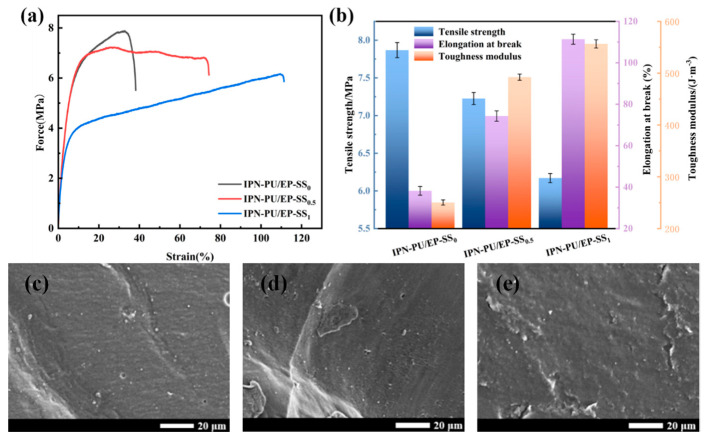
(**a**) Stress–strain curve of IPN-PU/EP-SS_x_; (**b**) Histogram of tensile strength, elongation at break, and toughness modulus; (**c**–**e**) Tensile fracture image of IPN-PU/EP-SS_0_, IPN-PU/EP-SS_0.5_, and IPN-PU/EP-SS_1_.

The stress–strain curves of IPN-PU/EPS-SS_0_, IPN-PU/EPS-SS_0.5_, and IPN-PU/EP-SS_1_ are exhibited in Figure 4a, while the tensile strength, elongation at break, and toughness modulus of the three samples are presented in Figure 4b. The tensile strength of IPN-PU/EP-SS_0_ reached 7.87 MPa. However, its tensile elongation and toughness modulus were only 38.22% and 250.32 J·m^−3^, respectively. As the density of dynamic disulfide bonds increased, the material’s tensile strength gradually decreased while the tensile elongation gradually increased. The tensile strengths of IPN-PU/EP-SS_0.5_ and IPN-PU/EP-SS_1_ were 7.23 MPa and 6.20 MPa, respectively. The bond energy of the C–C bond was 346 KJ·mol^−1^, while the bond energies of the C–S bond and S–S bond were 272 KJ·mol^−1^ and 251 KJ·mol^−1^, respectively. Consequently, the increase in the density of S–S bonds resulted in a reduction in the strength of the crosslinked network, which, in turn, led to a decline in the tensile strength of the material. As a damping material, IPN-PU/EP-SS_x_ is primarily applied in areas such as energy absorption and impact resistance. Consequently, in the evaluation of its mechanical properties, the elongation at break and toughness modulus become more critical considerations. Compared to IPN-PU/EP-SS_0_ and IPN-PU/EP-SS_0.5_, IPN-PU/EP-SS_1_ demonstrates a superior elongation at break and toughness modulus, with a maximum elongation at break of 111.39% and a toughness modulus of 556.53 J·m^−3^. Although its tensile strength is the lowest among the three samples, its overall mechanical performance remains the best of the three in the context of its application as a damping material. This indicates that the introduction of disulfide bonds can enhance the relevant performance of IPN as a damping material.

The microscopic fracture morphology of the IPN-PU/EP-SS_x_ tensile specimens is shown in Figure 4c–e. In comparison to IPN-PU/EP-SS_0.5_ and IPN-PU/EP-SS_1_, the fracture morphology of IPN-PU/EPS-SS_0_ was relatively flat, exhibiting a lack of discernible bar-shaped growth cracks and an evident groove texture. This observation suggests that the degree of forced inter-compatibility between polyurethane and epoxy resin was relatively low, indicating a two-phase separation structure may be present. With the increase in disulfide bond density, the roughness of the fracture surfaces of IPN-PU/EP-SS_0.5_ and IPN-PU/EP-SS_1_ was observed to increase. The fracture characteristics demonstrated the material’s ductility, indicating that plastic deformation occurred prior to failure. This result is consistent with the conclusions drawn from the tensile curves. The extent of plastic deformation varied depending on the density of disulfide bonds. These findings collectively demonstrate that the fracture mechanism incorporates characteristics of ductile fracture and is influenced by the internal structure of the material. When disulfide bonds are present within the interpenetrating networks, the material undergoes significant deformation before experiencing ductile failure. This also elucidates the phenomenon observed in the stress–strain curves, where an increase in the dynamic disulfide bond density enhances the toughness of the IPN-PU/EP-SSx.

### 3.4. DMA Characterization

**Figure 5:**
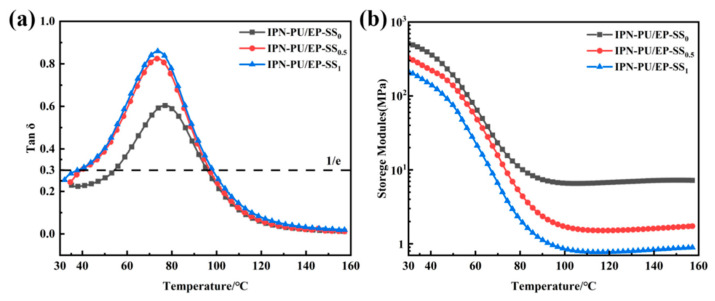
DMA curves of IPN-PU/EP-SS_x_: (**a**) temperature-loss factor; (**b**) temperature-storage modulus.

The temperature-loss factor (Tan δ) curves for IPN-PU/EP-SS_x_ are presented in Figure 5a. All three materials exhibited a broad damping peak, indicating that the polyurethane and epoxy have formed an interpenetrating network structure, achieving a favorable “forced intercapacitation” effect. The temperature corresponding to the damping peak on the temperature-loss factor curve is identified as the *T*_g_ of the material. The *T*_g_ of the IPN-PU/EP-SS_0_, IPN-PU/EP-SS_0.5_, and IPN-PU/EP-SS_1_ samples was found to be 76.2 °C, 73.2 °C, and 73.1 °C, respectively. These results demonstrate that the *T*_g_ decreases gradually with an increase in dynamic disulfide bond density. This can be attributed to the longer molecular chain segments of 2-ADPS compared to MOCA, with an increase in the free volume of the polymer and a corresponding reduction in the degree of constraint on molecular chain mobility, thus reducing the *T*_g_. The impact of dynamic disulfide bond density on the damping properties of the materials can also be obtained from Figure 5a. As the density of dynamic disulfide bonds increased, the damping performance of the material improved significantly. The loss factor of IPN-PU/EP-SS_1_ reached a maximum of 0.859, while the effective damping temperature range (temperature range of Tan δ ≥ 0.3) also reached a maximum value of 61.4 °C (refer to Table 2 for relevant data). Compared to IPN-PU/EP-SS_0_, IPN-PU/EP-SS_1_ showed a substantial enhancement in damping performance, with a 0.255 increase in dissipation factor and a 19.9 °C increase in the effective damping temperature range, approaching lower temperatures. As the temperature rose to or above room temperature, the dynamic disulfide bonds in the interpenetrating network underwent an exchange reaction, which accelerated with the temperature increase. During these reversible exchange reactions, the rearrangement of the topological network promoted the movement of the molecular chain, converting the mechanical energy into thermal energy and thereby improving the material’s damping performance.

The curves of the temperature-storage modulus (E’) of IPN-PU/EP-SS_x_ are shown in Figure 5b. The storage modulus of IPN-PU/EP-SS_x_ exhibited characteristics similar to the glass transition of thermoset polymers, where the material had a high storage modulus at low temperatures but decreased by two orders of magnitude as the temperature increased. This phenomenon can be attributed to the increased molecular chain movement and reversible exchange of dynamic disulfide bonds at elevated temperatures, leading to a reduction in storage modulus and gradual transition into the rubbery plateau region.

**Figure 6:**
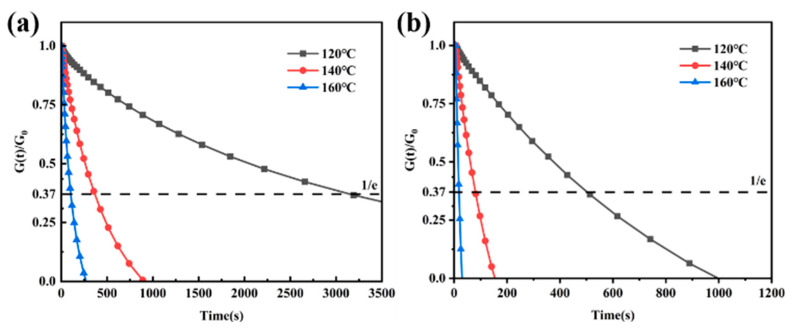
Stress relaxation curves at different temperatures: (**a**) IPN-PU/EP-SS_0.5_; (**b**) IPN-PU/EP-SS_1_.

The stress relaxation curves of IPN-PU/EP-SS_0.5_ and IPN-PU/EP-SS_1_ at 120 °C, 140 °C, and 160 °C are shown in Figure 6a,b, respectively. Both materials exhibited similar relaxation patterns, with the characteristic relaxation time (τ*) becoming progressively shorter with increasing temperature. This is due to the faster rate of dynamic disulfide bond exchange at higher external temperatures, which is manifested at the macroscopic level as a shorter τ* of the material. At each temperature, the τ* of IPN-PU/EP-SS_1_ was significantly shorter than that of IPN-PU/EP-SS_0.5_, with the τ* of IPN-PU/EP-SS_0.5_ at 160 °C being 102 s, while that of IPN-PU/EP-SS_1_ was only 18 s. This indicates that increasing the density of dynamic disulfide bonds significantly increases the frequency of their exchange, indirectly increasing molecular chain mobility and promoting the relaxation behavior of the material.

In order to further characterize the dynamic behavior of the material, the relaxation process was fitted using Maxwell’s model and Arrhenius’ equations (Equations (3)–(5)):

(3) $${\mathrm{Ln}\pi }^{*}={\ln\pi }_{0}+\frac{E_{a}}{\mathrm{RT}}$$

(4) $$H=G\tau^{*}$$

(5) $$G=\frac{E^{\prime }}{2\left(1+\nu \right)}$$

where E_a_ denotes the activation energy, R denotes the universal gas constant, and T is defined as the experimental temperature.

The slope of the fitted straight line represents the E_a_ of the material, with values of E_a_ being 123.88 kJ·mol^−1^ and 120.12 kJ·mol^−1^ for IPN-PU/EP-SS_0.5_ and IPN-PU/EP-SS_1_, respectively. The preceding results demonstrated that the augmentation in the density of dynamic disulfide bonds was accompanied by a reduction in the activation energy required for the chain reaction. This implied that the temperature required to initiate the reaction was diminished, thus rendering the chain exchange reaction more accessible.

### 3.5. Shape Memory Performance and Self-Healing Performance

**Figure 7:**
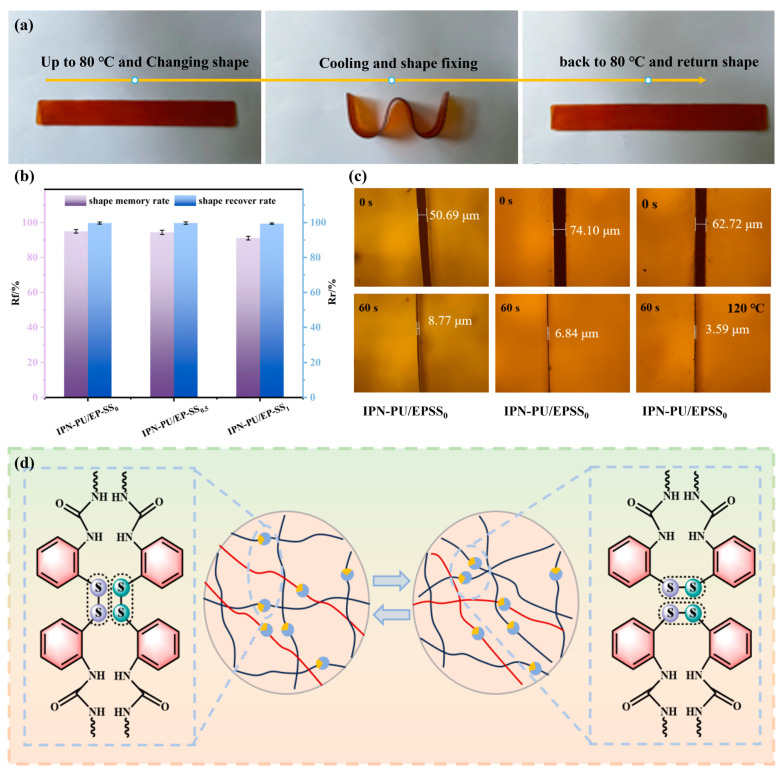
(**a**) The shape memory behavior; (**b**) Shape memory performance of IPN-PU/EP-SS_x_; (**c**) Self-repair ability of IPN-PU/EP-SS_x_; (**d**) Self-repair mechanism of dynamic disulfide bonds.

To visually demonstrate the shape memory properties of the sample, a rectangular strip specimen (160 mm × 20 mm × 1 mm) was fabricated using the synthesized material. The specimen was placed in an 80 °C (above the glass transition temperature, *T*_g_) water bath and heated for 1 min. Subsequently, it was rapidly twisted into a W-shape and cooled to room temperature to fix the configuration. The fixed specimen was then re-immersed in the 80 °C water bath to observe shape recovery. The entire process is documented in Figure 7a. *R*_r_ and *R*_f_ characterized the shape memory performance of IPN-PU/EP-SS_x_ and are presented in Figure 7b. The shape fixation rates of IPN-PU/EP-SS_0_, IPN-PU/EP-SS_0.5_, and IPN-PU/EP-SS_1_ were 95.0%, 94.4%, and 91.1%, respectively, while the shape recovery rates were 99.7%, 99.7%, and 99.4%, respectively. This indicates that IPN-PU/EP-SS_0_, IPN-PU/EP-SS_0.5_, and IPN-PU/EP-SS_1_ exhibited excellent shape memory properties, demonstrating high shape fixation and shape return rates. The temporary shape stored by the material was extremely temperature sensitive, and once the temperature was increased above *T*_g_, the material fully returned to its original shape. The dynamic exchange of disulfide bonds further increased the mobility of the polymer chains, facilitating the release of internal stresses and increasing the likelihood that the conformation of the polymer chains would return to its original shape under entropy-driven forces [41]. However, with increased disulfide bond density, the shape fixation rate of the materials showed a decreasing trend. This phenomenon was due to the different molecular configurations of 2-ADPS and MOCA and the weak disulfide bond energy. The increase in disulfide bond density sacrificed the strength of the hard segments in IPN-PU/EP, resulting in a lower shape fixation rate for the material.

Optical microscopy images of IPN-PU/EP-SS_1_, IPN-PU/EP-SS_0.5_, and IPN-PU/EP-SS_0_ before and after repair at 120 °C for 60 s comparison are presented in Figure 7c. The self-repair rate of the materials was determined by comparing the width of the scratches before and after repair. The self-repair rates of IPN-PU/EP-SS_0_, IPN-PU/EP-SS_0.5_, and IPN-PU/EP-SS_1_ were 82.69%, 90.77%, and 94.28%, respectively. Although IPN-PU/EP-SS_0_ does not contain disulfide bonds, it still exhibits a remarkably excellent self-healing efficiency. In this system, the polyurethane network primarily exists in a crosslinked state. However, incomplete crosslinking during the synthesis process results in residual linear or lightly crosslinked polyurethane segments. These segments retain sufficient flexibility to undergo rearrangement above the *T*_g_, thereby promoting the self-healing behavior of the material. Therefore, the movement of polyurethane molecular chains constitutes the fundamental mechanism of self-healing in IPN-PU/EP-SS_0_. As the density of disulfide bonds increases, IPN-PU/EP-SS_0.5_ and IPN-PU/EP-SS_1_ demonstrate even higher self-healing efficiency. This indicates that the incorporation of disulfide bonds enhances the inherent self-healing capability of the system. On one hand, disulfide bonds possess a reversible exchange nature; on the other hand, they act as connections within the molecular chains, promoting the mobility of the polyurethane chains. The reversible exchange mechanism of dynamic disulfide bonds facilitates molecular chain mobility and topological network rearrangement, serving as a pivotal factor in enhancing the self-healing capability of IPN systems.

Disulfide bonds are dynamic covalent bonds capable of reversible cleavage and recombination, and thus, the self-healing mechanism of polymers containing disulfide bonds primarily relies on the reversible breaking and reforming of these bonds under specific stimuli. The fundamental principles involved in the self-healing behavior of such polymers are illustrated in Figure 7d. Under external stimuli, S–S bonds can dissociate into thiyl radicals (R-S•), which are highly reactive and can recombine to form new disulfide bonds, thereby enabling the polymer network to self-repair. When the material is subjected to mechanical stress, microcracks or fractures may develop within the polymer network, and the disulfide bonds near the crack surfaces undergo cleavage, generating thiol groups. Continued application of external force will lead to complete material fracture. If the external force is removed and the cracked material is placed in a free environment, the molecular chains become frozen at low external temperatures (≤*T*_g_), preventing the exchange of dynamic disulfide bonds. However, as the temperature increases (>*T*_g_), the dynamic disulfide bonds are activated, and adjacent thiyl radicals recombine to form new disulfide bonds, effectively bridging the cracks and restoring the integrity of the polymer network, thereby facilitating the self-healing process. This characteristic of disulfide bonds promotes the rapid movement of molecular segments at elevated temperatures, allowing for the reconnected disulfide bonds to bond the fractured material together, thus repairing the generated cracks [42]. As a damping material, the heat generated by the damping loss can facilitate the topological rearrangement of the internal dynamic disulfide bonds in the PU/EP-IPN, thereby accelerating the self-healing process. This property of the disulfide bonds contributes to enhanced self-repairing capability of the PU/EP-IPN under mechanical stress, making it a promising candidate for applications requiring durability and longevity in dynamic environments.

## 4. Conclusions

In conclusion, a series of novel PU/EP-IPNs that exhibited enhanced properties compared to their individual components were synthesized and characterized. The incorporation of dynamic disulfide bonds significantly improved the shape memory and self-healing properties of the polymeric system. Quantitative evaluation revealed remarkable shape retention characteristics, achieving fixation efficiency of 95.0% and recovery ratios reaching 99.7%, demonstrating the materials’ ability to return to their original shape upon heating above the *T*_g_. The effective damping temperature range of the material reached 61.4 °C, with a loss factor of 0.859, indicating improved damping performance as the density of disulfide bonds in the network increased. Additionally, the establishment of an interpenetrating polymer network architecture between polyurethane and epoxy components contributed to significant improvements in both mechanical strength and thermomechanical stability.

These novel PU/EP-IPNs exhibit considerable potential as a damping material with self-healing capabilities, providing a robust foundation for future research in this field. The findings underscore the importance of optimizing the balance between disulfide bond density and mechanical strength to maximize the performance of these materials.

## Figures and Tables

**Table 1:** The specific compositions of the samples.

Samples	Polyurethane Prepolymers (g)	E51 (g)	2-ADPS (g)	MOCA (g)	Cashew Phenol (g)
IPN-PU/EP-SS_0_	10.1	9.0	/	5.3	2.24
IPN-PU/EP-SS_0.5_	10.1	9.0	2.5	2.6	2.22
IPN-PU/EP-SS_1_	10.1	9.0	5.1	/	2.22

**Table 2:** DMA data of IPNs-PU/EP-SS_x_.

Sample	Damping Temperature Range (°C) tan δ ≥ 0.3	ΔT at tan δ ≥ 0.3/°C	tan δ (max)	*T*_g_ (°C)
IPNs-PU/EP-SS_0_	54.3–95.8	41.5	0.604	76.2
IPNs-PU/EP-SS_0.5_	39.7–97.0	57.3	0.824	73.2
IPNs-PU/EP-SS_1_	37.0–98.4	61.4	0.859	73.1

## Data Availability

The original contributions presented in this study are included in the article. Further inquiries can be directed to the corresponding authors.
